# Mechanized wide narrow row densification configuration enhances japonica rice yield and head rice quality in Northern China

**DOI:** 10.3389/fpls.2026.1727060

**Published:** 2026-02-04

**Authors:** Liqiang Dong, Maoxing Song, Tiexin Yang, Liang Ma, Yuedong Li, Yi Huang

**Affiliations:** 1 Liaoning Academy of agricultural sciences, Liaoning Rice Research Institute, Shenyang, Liaoning, China; 2 Tangshan Academy of Agricultural Sciences, Tangshan, Hebei, China; 3 Comprehensive Affairs Service Center, Boluopu Town, Dashiqiao, Liaoning, China

**Keywords:** fine rice powder, mechanized rice production, rice quality, rice yield, row and hill spacing modes

## Abstract

**Objective:**

Planting configuration is an important factor in regulating rice yield and quality. This study aimed to elucidate the effects of mechanized transplanting configuration on yield formation and grain quality in northern China.

**Methods:**

A two-year field experiment (2022-2023) was conducted using the japonica cultivar Liaojing 419, comparing four planting modes: local farmer’s method (LFM, equal row spacing), conventional densification (CDM), narrow-row densification (NDM), and wide-narrow row densification (WNDM). Measurements included yield and its components, biomass accumulation, soluble sugar content in grains, processing quality (head rice yield), cooking and eating quality, and starch granule morphology of milled rice powder.

**Results:**

The order of grain yield is WNDM>NDM>CDM>LFM, although NDM has the highest dry matter mass and productive panicles, WNDM has the highest product of total grain number (/m^2^) and thousand grain weight, which determines rice yield. LFM has the best processing quality (67.66 and 68.66 in head rice rate, respectively), appearance quality, taste quality and produced milled rice powder with the smoothest particle surfaces, followed by WNDM. WNDM led to the highest soluble sugar content in grains at 15, 25and 35 days after anthesis and highest head rice yield (7,895.51 and 8,066.34 kg/ha). The soluble sugar content in grains at 25and 35 days were extremely significantly (p<0.01) positively correlated with head rice yield.

**Conclusion:**

The mechanized wide-narrow row densification configuration presents a balanced strategy that enhances grain yield and milling quality while maintaining acceptable eating quality. It offers a promising planting method for improving overall productivity in mechanized japonica rice systems in northern China.

## 1 Introduction

As the world’s largest rice (*Oryza sativa* L.) producer, China has produced more than 200 million tons of rice for 14 consecutive years (Chen et al., 2025). Notably, northern japonica rice production areas, with unique climate and soil conditions, have demonstrated excellent performance in terms of the taste, appearance, and other quality characteristics of rice (Yan et al., 2025), and the market share and demand for high-quality rice in China have increased annually (Zhou et al., 2019; Chen et al., 2025). However, with population growth and the gradual reduction in arable land, increasing rice yields while maintaining or increasing the rice quality in areas with limited land resources has become an important issue among agricultural researchers (Li et al., 2023; Song et al., 2025).

The row and hill spacing configuration, as a key agronomic measure in rice production, directly affects rice growth and development, population establishment, and ultimately the yield and quality (Zheng et al., 2021). A high yield and high-quality row and hill spacing configuration can facilitate the coordination of the development of populations and individuals, the effective optimization of the light distribution and ventilation conditions for rice populations, and the utilization of different levels of light resources, thereby affecting physiological processes such as the photosynthesis efficiency, nutrient absorption and utilization efficiency of plants (Xiong et al., 2021, 2022) to increase yields. In recent years, with the increase in the scale of intensive rice production and the in-depth integration of agricultural machinery and agronomy, completely mechanized seedling cultivation, transplanting, and harvesting processes have been established in northern japonica rice production areas, providing a solid foundation for high-quality rice development (Zhu et al., 2021; Li et al., 2023). The demand for agricultural development and technological innovation is promoting the transformation of rice production in these regions toward a production mode in which cost savings and high-quality production are combined.

However, the conventional equal-row spacing configuration struggles to reconcile these multidimensional objectives, as its uniform architecture often leads to inefficient light interception (e.g., lower photosynthetic active radiation capture compared to optimized canopies), exacerbated inter-plant competition, and compromised stress resilience (Xiong et al., 2021). Although the conventional equal-row spacing configuration (e.g., 30 × 18 cm) is widely implemented in the production of northern japonica rice, the yield potential of populations cannot be fully realized (Dong et al., 2023). It is particularly necessary to explore more efficient ways to configure the plant spacing (Zhu et al., 2015; Li et al., 2015). Traditional densification cultivation mode only aims to increase the transplanting density through the reduction in the distance between plants, thereby increasing the number of basic seedlings to promote an increase in the number of spikes per unit area and in turn increasing the rice yield. Excessive and unoptimized densification can lead to limited growth space for individual plants, which may cause issues such as insufficient photo-assimilate supply, incomplete grain filling, uneven starch distribution, and ultimately reduced rice quality (Zhou et al., 2019; Deng et al., 2022; Chen et al., 2023; Yan et al., 2025). There is an urgent need to develop a plant spacing configuration model that balances yield increase and optimization to meet the needs of modern northern japonica rice production.

On the basis of the above findings, this study focused on the main cultivated japonica rice varieties and commercialized machine transplanting methods in northern China. The conventional plant spacing mode (local farmers’ mode (LFM); 30 × 18 cm) and three densification modes (conventional densification mode (CDM), narrow row densification mode (NDM), and wide–narrow row densification mode (WNDM)) were investigated. The objective of this study was to systematically analyze the regulatory effects of the plant spacing configuration on rice yield formation, processing quality, appearance quality, and starch characteristics and to elucidate the mechanisms through which grain-filling material sources influence the rice yield and quality under various densification cultivation modes. This study was to provide an evaluation of mechanized row and hill spacing configuration as a transformative strategy to resolve the long-standing trade-off between yield enhancement and quality preservation in northern japonica rice systems.

## 2 Materials and methods

### 2.1 Experimental varieties and locations

The tested variety was Liaojing 419, which was provided by the Liaoning Rice Research Institute. Liaojing 419 is the main variety used for large-scale production in the rice-growing area of central Liaoning with a potential yield of 13500 kg/hm^2^ in Liaoning Province.

### 2.2 Experimental design

In the field experiment, sowing was conducted on April 13 and April 14, 2022 and 2023; transplanting was performed on May 15 and May 16; and mechanized harvesting was carried out on October 5 and October 7 of the two years. The following four planting modes were used for treatment: LFM: Local farmer cultivation mode (row and hill spacing of 30 cm × 18 cm); CDM: conventional densification mode (row and hill spacing of 30 cm × 14 cm); NDM: narrow row densification mode (row and hill spacing of 25 cm × 17 cm); WNDM: wide narrow row densification mode (row and hill spacing of 36 + 14 cm × 16 cm). The experiment was conducted using a randomized block design with three replications. All plots were 100 m long with 48 rows. Plot width and total area were 14.4 m and 1440 m² for LFM and CDM (30 cm row spacing), and 12.0 m and 1200 m² for both NDM (25 cm row spacing) and WNDM (36cm+14cm wide-narrow row spacing).

In this study, fertilizer application rate is consistent with local high-yield recommendations (Dong et al., 2023, 2024) and was chosen to eliminate fertilizer limitation as a variable, ensuring that observed differences in growth and yield were primarily attributable to the planting configuration treatments. he application rate of pure N (slow-release urea) is 300.0 kg/hm^2^ (base fertilizer: tiller fertilizer: panicle fertilizer ratio = 6:3:1); the application rate of pure P (diammonium phosphate) is 69.0 kg/hm^2^ (base fertilizer); and the application rate of pure K (potassium chloride) is 75.0 kg/hm^2^ (base fertilizer: panicle fertilizer ratio = 1:1). Field management methods were employed in the experimental area (Dong et al., 2023).

### 2.3 Measured parameters and methods

#### 2.3.1 Growth dynamics

Stem and tiller dynamics: 20 hills stem and tiller number survey were conducted every 7 days from the time of rice transplanting to the full heading stage, and the number of stems at the transplanting, jointing, heading, full heading, and maturity stages was recorded in the same site of each plot.

#### 2.3.2 Rice yield and yield components

Rice yield: After the maturity period, a Kubota 988 harvester (Kubota, Suzhou, China) was employed for mechanized harvesting. After removing missing yield data from the sample, the yield was calculated on the basis of a moisture content of 14.5%.

Rice yield components: Before mechanized harvesting, 10 plants with consistent growth characteristics were selected from each plot for indoor seed testing. The number of productive panicles, number of full grains per panicle, seed setting rate, and thousand-seed weight were measured.

The total number of full grains (/m^2^) can be calculated as productive panicles × number of full grains per panicle.

#### 2.3.3 Biomass accumulation

To assess traits at the population level, a standardized sampling of representative plants was performed at jointing, heading, and maturity stages. In each plot, four plants exhibiting growth consistent with the plot average (based on 2.3.1 stem and tiller number) were selected. This method, aimed at capturing the central tendency of the population rather than its full variance, is a common practice for destructive sampling in field plot experiments. Subsequent measurements of leaf area index (leaf weight method), biomass, and stem sheath NSC content (Ren et al., 2022 method) were performed on these samples.

#### 2.3.4 Soluble sugar and starch accumulation

At the heading stage, 50 stems with similar flowering periods and field plant heights were labeled with tags. At 15, 25, and 35 days after flowering (DAF), the leaves and grains of 10 labeled plants were rendered inactive at 105 °C for 0.5 hours, dried at 80 °C to a constant weight, crushed, and sieved through a 100 mesh sieve. A 0.1-g sample was weighed into a 10-mL centrifuge tube, after which 8 mL of an 80% ethanol solution was added. After cooling, the samples were centrifuged three times. The supernatants were combined to determine the content of soluble sugars, and the residue was used for starch analysis. The anthrone colorimetric method was applied to determine the contents of soluble sugars and starch (Wang et al., 2014).

Sugar/starch accumulation can be calculated as soluble sugar/starch content (%) × biomass (g).

#### 2.3.5 Rice quality

Dried rice grains were subjected to rice cooking quality testing after storage at room temperature for 3 months. After wind-based selection, the rice was ground using a brown rice mill (FC2K, Yamamoto, Japan) and milled rice mill (VP-32, Yamamoto, Japan). Then, the brown rice rate (%), milled rice rate (%), head rice rate (%), chalkiness rate (%), and chalkiness degree were determined three times for each sample according to the national standard (GB/T17891–2017 High Quality Rice) (Standardization Administration of the People’s Republic of China, 2017).

The head rice yield (an economic output, kg/ha) can be calculated as grain yield (kg/ha) × head rice rate (%).

#### 2.3.6 Rice taste value

A total of 30 g of head rice was weighed and placed in a steel jar. The mixture was washed until the water was almost clear. Thereafter, water was added at a ratio of 1.0:1.3, and the samples were covered with filter paper and soaked for 30 minutes. The steel jar containing soaked rice was placed in a steamer, and the rice was steamed for 30 minutes, after which the steamed rice was kept warm for 10 minutes. The rice was gently stirred, covered with filter paper and cooled for 20 minutes. The samples were removed and covered with a matching steel cover and allowed to cool naturally for 90 minutes. Finally, a rice sample weighing 8.00 ± 0.05 g was collected. A rice taste meter (STA-1B, Satake, Japan) was employed to measure the appearance, hardness, viscosity, and taste of the steamed rice. Each sample was measured in triplicate.

#### 2.3.7 Preparation of finely ground powder

After processing (the milled rice was naturally dried to a moisture content of 14%), the rice was ground into powder using a milling machine at a grinding speed of 1000 r/min and a grinding time of 30 seconds. The ground rice was sieved twice through a 100 mesh screen to obtain a fine powder, which was stored at 4 °C for future use.

#### 2.3.8 Electron microscopy scanning of finely ground particles

The fine rice powder was quantitatively analyzed using a field emission scanning electron microscope (Regulus 8100, HITACHI, Japan) after gold spray coating, and the microstructure was observed. After drying, the sample was evenly affixed to the sample disk, a gaseous secondary electron detector was employed to observe the sample at 5 kV, and photos were obtained under 500 times, 2000 times, and 5000 times magnification for analysis.

### 2.4 Statistical analysis

Excel (2020, Microsoft, Redmond, WA, USA) was used for data organization, SPSS (22.0; SPSS Inc., Chicago, IL, USA) was used for data difference analysis, and Origin (Origin Lab, Hampton, MA, USA) was used for data plotting. All data are expressed as the mean ± standard deviation, with three replicates. Multiple comparisons of means were based on Fisher’s least significant difference (LSD) test at 5% and 1% significance level unless stated otherwise.

## 3 Results

### 3.1 Grain yield and yield components

**Figure 1:**
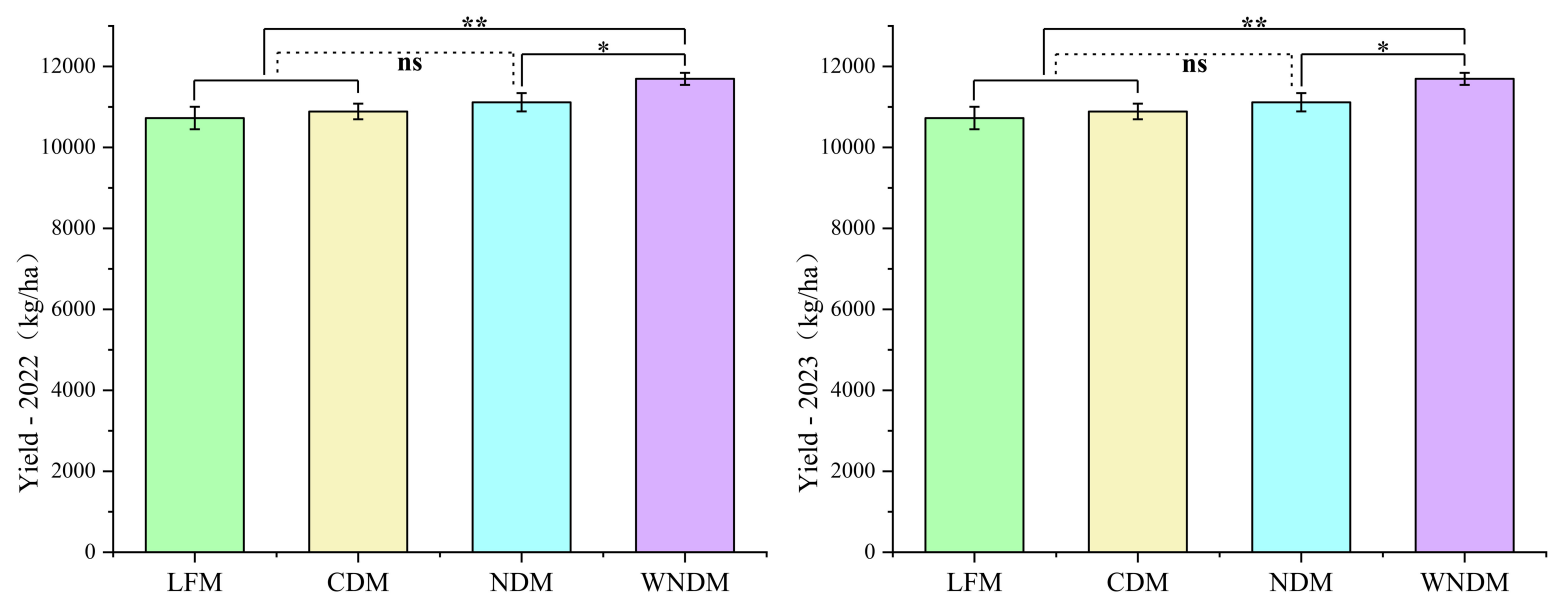
Grain yield. A one-way analysis of variance (ANOVA) followed by least significant difference (LSD) multiple range test was per-formed to evaluate the statistical differences among the treatments per each parameter measured. * indicates the P < 0.05, ** indicates the P < 0.01, ns indicates no significant.

To determine the influence of the plant spacing configuration on rice traits, we first aimed to determine the characteristics of grain yield and yield components through a field experiment involving plots with different planting modes. The actual harvest quality of grains is one of the key indicators for measuring the effectiveness of rice production. In the field, the actual yield trends under the four methods over two years period were consistent (**Figure 1**), with the LFM method exhibiting the lowest average yield, which is 1.4%, 3.0%, and 8.1% lower than those under the CDM, NDM, and WNDM methods, respectively. The yields of the WNDM method were the highest over two years period, at 11693.56 and 11823.59 kg/ha, which are significantly (p<0.05) greater than those of the NDM method and extremely significantly (p<0.01) greater than those of the LFM and CDM methods. Under the WNDM method, the rice yield increased significantly (p<0.05).

Yield composition factors determine the theoretical yield of rice per unit area. The Year × Mode interaction was significant for productive panicle number, full grain number, thousand seed weight, and total grain number (P < 0.05 or P < 0.01), the seed setting rate showed no significant interaction (P > 0.05). According to the analysis of the yield composition factors under the four methods (**Table 1**), there were significant differences in the productive number of panicles between the years and methods, with the LFM providing a significantly smaller value than the other methods and the NDM exhibiting the highest productive number of panicles. The number of full grains and the seed setting rate of the CDM, NDM, and WNDM were significantly (p<0.05) lower than those of the LFM. The thousand-seed weight of the LFM was the highest, with values of 25.04 and 25.18 g over the two year, which are significantly (p<0.05) greater than those of the CDM and NDM in 2022 and significantly (p<0.05) greater than that of the NDM in 2023. The total number of grains of the LFM was the lowest, and the value was significantly (p<0.05) lower than that of the NDM and WNDM over two years period, which is consistent with the production trend.

**Table 1:** Yield components.

Year	Method	Productive panicle number (/m^2^)	Full grain number	Seed setting rate (%)	Thousand seed weight (g)	Total grain number(/m^2^)
2022	LFM	358.35 ± 3.18Cc	124.63 ± 0.72Aa	91.43 ± 0.24Aa	25.04 ± 0.10Aa	44659.27 ± 314.14Bc
	CDM	414.08 ± 3.73Bb	113.15 ± 0.44Bb	86.06 ± 0.25Cc	24.33 ± 0.10Bc	46851.29 ± 176.65Ab
	NDM	435.65 ± 3.88Aa	109.74 ± 0.17Cc	83.06 ± 0.74Dd	24.58 ± 0.13ABbc	47806.85 ± 62.35Aa
	WNDM	430.29 ± 3.86Aa	111.07 ± 0.56BCc	87.80 ± 0.25Bb	24.90 ± 0.12Aab	47791.18 ± 244.18Aa
2023	LFM	370.79 ± 0.44Dd	122.87 ± 0.82Aa	93.41 ± 1.07Aa	25.18 ± 0.22Aa	45560.25 ± 293.23Bb
	CDM	422.42 ± 0.06Cc	109.19 ± 0.70Bb	86.57 ± 0.66BCc	25.03 ± 0.16Aab	46125.83 ± 308.39Bb
	NDM	441.65 ± 0.22Aa	109.03 ± 1.06Bb	84.02 ± 0.41Cd	24.44 ± 0.26Ab	48151.06 ± 438.45Aa
	WNDM	433.41 ± 0.16Bb	110.76 ± 0.52Bb	88.30 ± 0.66Bb	25.07 ± 0.04Aa	48005.00 ± 193.15Aa
	Y	**	**	**	**	ns
	M	**	**	**	**	**
	Y×M	**	*	ns	**	*

Data in the table are means ± standard deviation, with three replicates. Different uppercase letters and lowercase letters indicate significant difference at P < 0.05 and P < 0.01 probability level within a column in a year, respectively. A two-way ANOVA was used to test the effects of mode, year, and their interactions. Y·indicates the year, M·indicates the mode,·Y × M·indicates the interaction between year and mode. * indicates the P < 0.05, ** indicates the P < 0.01, ns indicates no significant.

### 3.2 Dynamic tillering during the growth period

The number of tillers is the most important factor influencing the rice yield. The Year × Mode interaction was not significant (P > 0.05) for all stages. The trend of the annual changes in the number of tillers was consistent (**Table 2**), which indicates that as the growth period progressed, the number of tillers first increased but then decreased until it eventually stabilized. The number of stems increased rapidly at the jointing stage, with a peak in the number of tillers at the heading stage. Afterward, small tillers died, causing a decrease in the number of stems at the full heading stage until the number of stems of the population stabilized after maturity. The processing behavior revealed the order of NDM>WNDM>CDM>LFM. Compared with that under the other treatments, the number of stems under the LFM treatment was significantly (p<0.05) smaller at all growth stages, and the number of stems decreased by 56.74–78.31/m^2^ and 52.29–71.20/m^2^ at the maturity stage over two years period, which affected the yield.

**Table 2:** Tiller formation.

Year	Method	Transplanting (/m^2^)	Jointing (/m^2^)	Heading (/m^2^)	Full Heading (/m^2^)	Maturity (/m^2^)
2022	LFM	67.32 ± 1.5Bb	340.09 ± 7.65Bb	421.52 ± 9.46Bb	398.25 ± 7.71Bb	361.75 ± 7.15Cc
	CDM	82.55 ± 1.12Aa	447.23 ± 6.21Aa	534.94 ± 7.38Aa	503.4 ± 5.82Aa	418.48 ± 2.43Bb
	NDM	82.66 ± 1.69Aa	449.43 ± 9.07Aa	547.1 ± 11.08Aa	505.39 ± 7.11Aa	440.05 ± 2.54Aa
	WNDM	83.54 ± 0.93Aa	436.24 ± 4.76Aa	520.18 ± 5.72Aa	489.93 ± 6.64Aa	434.36 ± 2.21Aa
2023	LFM	66.58 ± 1.49Bb	339.36 ± 7.73Bb	425.98 ± 7.06Bc	404.45 ± 9.05Bc	374.52 ± 5.23Bc
	CDM	81.98 ± 1.12Aa	447.02 ± 6.29Aa	540.38 ± 4.94Aab	506.99 ± 6.95Aab	426.82 ± 6.14Ab
	NDM	82.78 ± 1.68Aa	457.67 ± 9.14Aa	550.39 ± 3.69Aa	516.71 ± 10.5Aa	445.72 ± 5.39Aa
	WNDM	84.95 ± 0.93Aa	454.29 ± 4.85Aa	523.52 ± 8.27Ab	494.32 ± 5.47Ab	437.81 ± 5.91Aab
	Y	ns	ns	ns	ns	**
	M	**	**	**	**	**
	Y×M	ns	ns	ns	ns	ns

Data in the table are means ± standard deviation, with three replicates. Different uppercase letters and lowercase letters indicate significant difference at P < 0.05 and P < 0.01 probability level within a column in a year, respectively. A two-way ANOVA was used to test the effects of mode, year, and their interactions. Y·indicates the year, M·indicates the mode,·Y × M·indicates the interaction between year and mode. * indicates the P < 0.05, ** indicates the P < 0.01, ns indicates no significant.

### 3.3 Biomass accumulation

**Figure 2:**
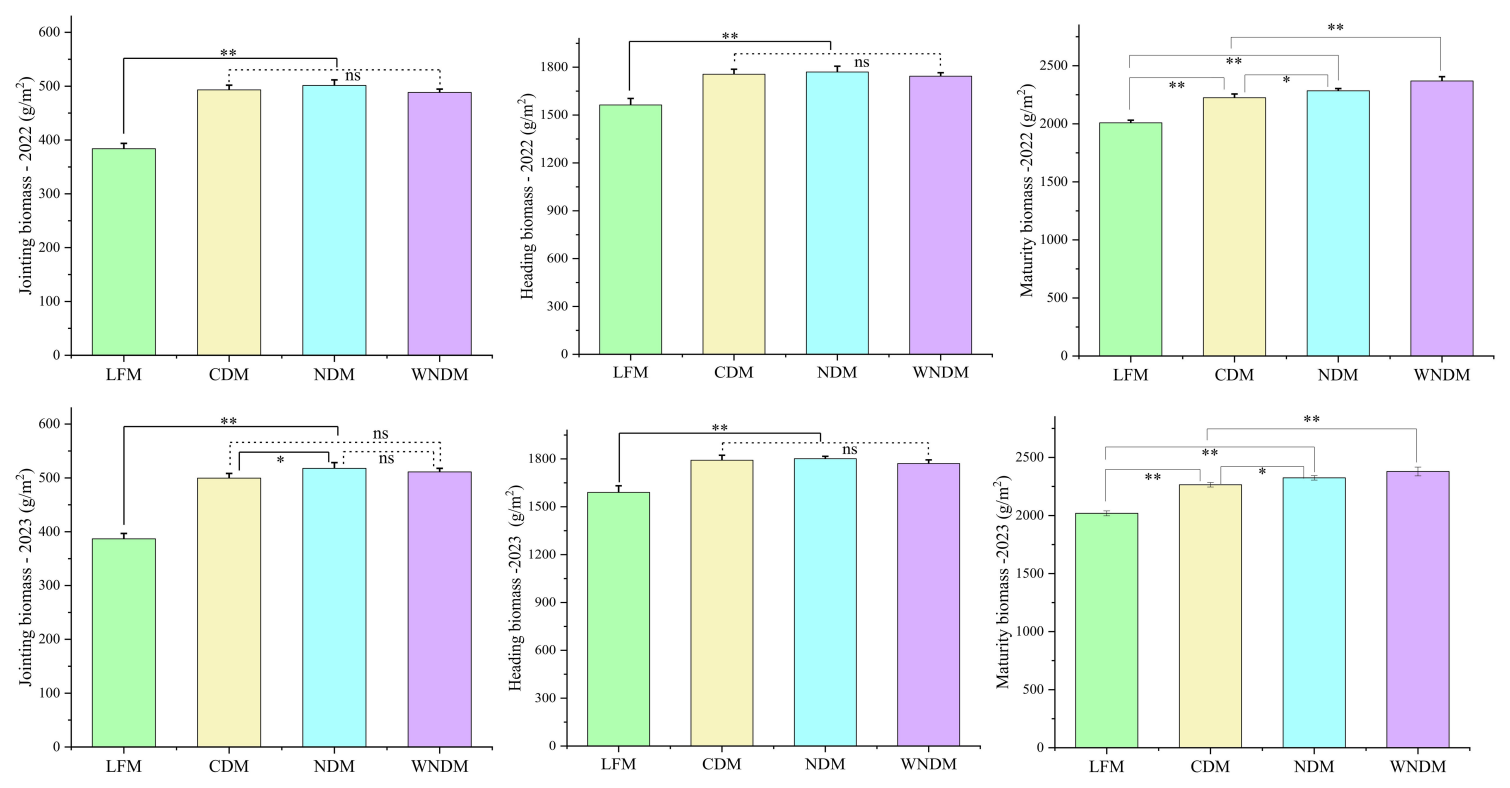
Biomass of the rice population. A one-way analysis of variance (ANOVA) followed by least significant difference (LSD) multiple range test was per-formed to evaluate the statistical differences among the treatments per each parameter measured. * indicates the P < 0.05, ** indicates the P < 0.01, ns indicates no significant.

Biomass is the source of assimilates for rice grain yield and quality formation, and the biomass accumulation trend was consistent between years, with a slight increase in 2023 compared with that in 2022 (**Figure 2**). The LFM yielded the lowest biomass across all the growth stages, and the value was significantly (p<0.05) lower than that of the three densification methods. The highest biomass was obtained at both the jointing and heading stages under the NDM method, which is significantly (p<0.05) higher than that under the CDM method in 2023. The highest levels were observed at the mature stage under WNDM method, with values of 2368.50 and 2379.44 g/m^2^ over two years period, which are significantly (p<0.05) higher than those under the other methods.

### 3.4 Accumulation of carbohydrates during the grain-filling period

The transport of photosynthetic compounds produced by rice source organs in the form of sucrose through the phloem to the grains determines the quality of rice. The Year × Mode interaction significant (P < 0.05) for soluble sugar in rice leaf at 35d and extremely significant (P < 0.01) for soluble sugar in grain at 35d. There were significant differences in the soluble starch and soluble sugar contents per unit area of leaves and grains between 15 and 25 DAF. The soluble starch and soluble sugar contents in the leaves were highest under the NDM method and lowest under the LFM method. As the post-flowering filling process progressed, the soluble starch content in grains increased rapidly, with the value under the WNDM method significantly (p<0.05) higher than those under the CDM and NDM methods and extremely significantly (p<0.01) higher than that under the LFM method. The WNDM method resulted in the highest soluble sugar contents in grains at 15, 25, and 35 days, with those at 25 and 35 days significantly (p<0.05) greater than those of the CDM and NDM methods. The CDM and NDM methods resulted in a decrease in the soluble sugar content in grains at 35 days compared with that at 25 days, indicating that the effective supply of assimilates under both methods at the late stage of grain filling was lower than that under the WNDM and LFM methods at the population level.

### 3.5 Processing quality and head rice yield

**Figure 3:**
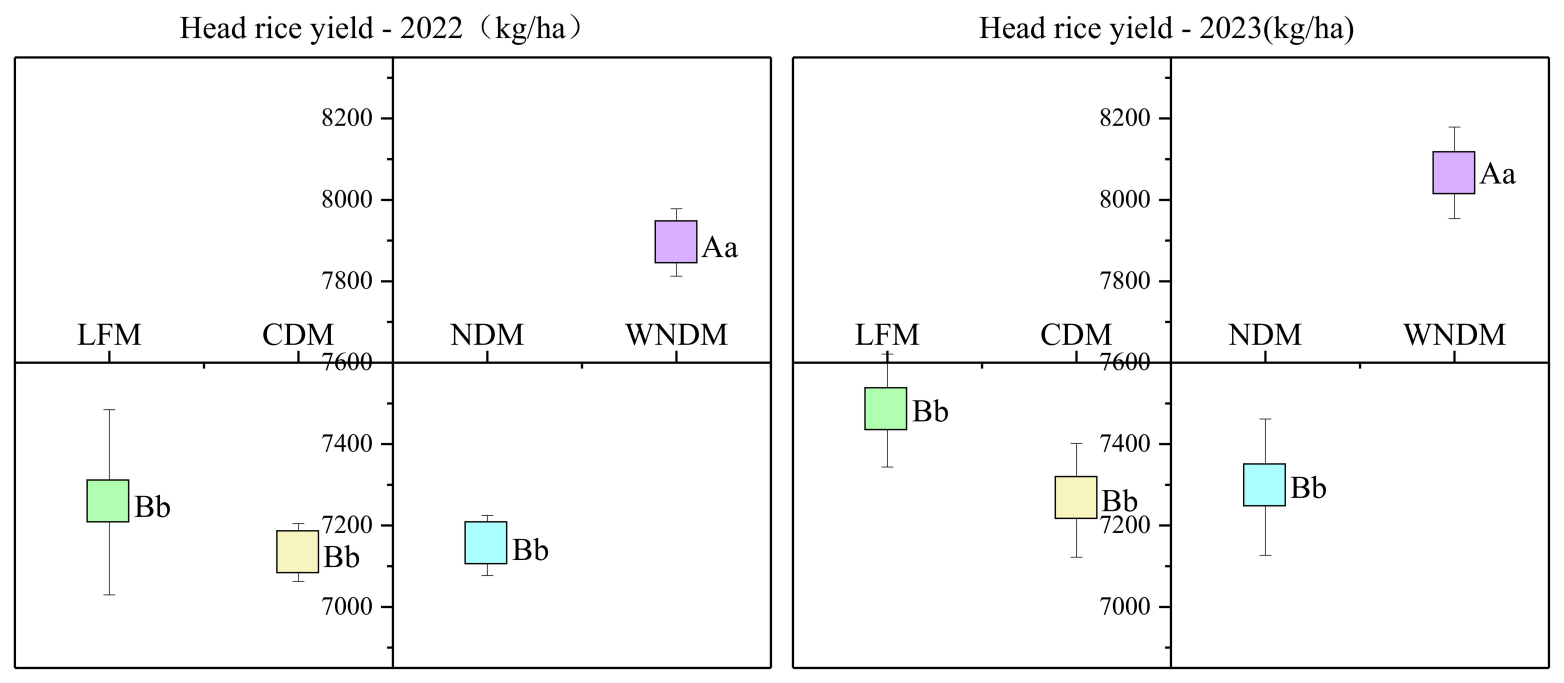
Head rice yield. A one-way analysis of variance (ANOVA) followed by least significant difference (LSD) multiple range test was per-formed to evaluate the statistical differences among the treatments per each parameter measured. Different uppercase letters and lowercase letters indicate significant difference at P < 0.05 and P < 0.01 probability level in a year, respectively.

The processing quality is an important indicator for evaluating the grain-to-rice ratio. The Year × Mode interaction was not significant (P > 0.05) for brown rice rate, milled rice rate, head rice rate and chalky grain rate, significant Year × Mode interaction was observed for chalkiness degree (P < 0.05). The measurement and analysis of the processing quality (**Table 3**) and the head rice yield (**Figure 3**) under the different plant spacing configurations revealed consistent interannual variation trends. There was no significant (p>0.05) difference in the rates of brown and milled rice between the different methods. The head rice rate indicated the order of LFM > WNDM > CDM > NDM. The LFM method provided two years head rice rate of 67.66% and 68.66%, which are significantly (p<0.05) higher than those of the NDM method. Both the chalkiness rate and chalkiness degree were highest under the NDM method and lowest under the LFM method. In 2023, the chalkiness rate of the NDM method was significantly (p<0.05) higher than that of the CDM method and extremely significantly (p<0.01) greater than those of the LFM and WNDM methods. Moreover, the chalkiness degree of the NDM method was significantly (p<0.05) greater than those of the other methods. The head rice yield of the WNDM method was significantly (p<0.05) greater than those of the other methods, with two years head rice yields of 7895.51 and 8066.34 kg/ha. There was no significant (p>0.05) difference between the LFM, CDM, and NDM methods.

**Table 3:** Processing quality.

Year	Method	Brown rice rate (%)	Milled rice rate (%)	Head rice rate (%)	Chalky grain rate (%)	Chalkiness degree
2022	LFM	81.37 ± 0.71Aa	72.52 ± 1.39Aa	67.66 ± 2.07Aa	5.64 ± 0.26Ab	1.89 ± 0.08Ab
	CDM	81.03 ± 0.67Aa	71.55 ± 1.39Aa	65.52 ± 0.69Aab	5.93 ± 0.26Aab	1.94 ± 0.03Aab
	NDM	80.86 ± 0.71Aa	71.43 ± 1.39Aa	64.33 ± 0.69Ab	6.23 ± 0.26Aa	2.03 ± 0.03Aa
	WNDM	81.14 ± 2.09Aa	72.14 ± 1.39Aa	67.52 ± 0.69Aa	5.83 ± 0.26Aab	1.91 ± 0.03Ab
2023	LFM	82.16 ± 0.70Aa	72.14 ± 1.39Aa	68.66 ± 1.25Aa	6.87 ± 0.26Bb	2.44 ± 0.08Bb
	CDM	80.64 ± 2.10Aa	71.23 ± 1.39Aa	65.75 ± 1.24Aab	7.37 ± 0.26ABb	2.50 ± 0.03Bb
	NDM	80.62 ± 0.70Aa	71.5 ± 1.39Aa	65.2 ± 1.52Ab	8.17 ± 0.26Aa	2.77 ± 0.03Aa
	WNDM	81.00 ± 0.70Aa	72.14 ± 1.39Aa	68.22 ± 0.98Aa	6.97 ± 0.26Bb	2.49 ± 0.03Bb
	Y	ns	ns	ns	**	**
	M	ns	ns	**	**	**
	Y×M	ns	ns	ns	ns	*

Data in the table are means ± standard deviation, with three replicates. Different uppercase letters and lowercase letters indicate significant difference at P < 0.05 and P < 0.01 probability level within a column in a year, respectively. A two-way ANOVA was used to test the effects of mode, year, and their interactions. Y·indicates the year, M·indicates the mode,·Y × M·indicates the interaction between year and mode. * indicates the P < 0.05, ** indicates the P < 0.01, ns indicates no significant.

### 3.6 Processing quality correlation

**Figure 4:**
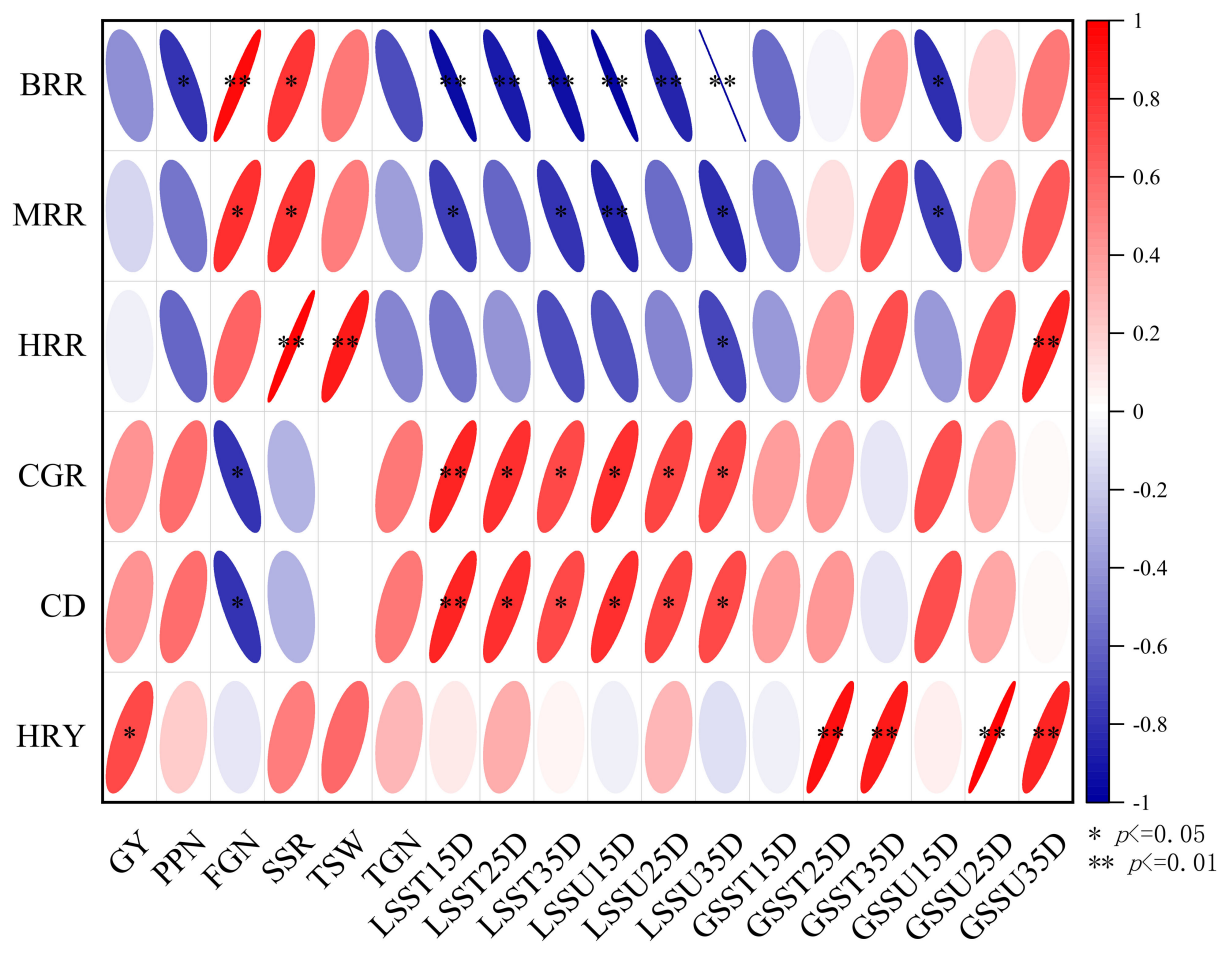
Correlations between the processing quality and agronomic traits. BBR is brown rice rate, MRR is milled rice rate, HRR is head rice rate, CGR is Chalky grain rate, CD is Chalkiness degree, HRY is head rice yield, GY is grain yield, PPN is productive panicles number, FGN is full grains number, SSR is seed setting rate, TSW is thousand seeds weight, TGN is total grains number, LSST15D is leaf soluble starch content at 15 day after anthesis, LSST25D is leaf soluble starch content at 25 day after anthesis, LSST35D is leaf soluble starch content at 35 day after anthesis, LSSU15D is leaf soluble sucrose content at 15 day after anthesis, LSSU25D is leaf soluble sucrose content at 25 day after anthesis, LSSU35D is leaf soluble sucrose content at 35 day after anthesis, GSST15D is grain soluble starch content at 15 day after anthesis, GSST25D is grain soluble starch content at 25 day after anthesis, GSST35D is grain soluble starch content at 35 day after anthesis, GSSU15D is grain soluble sucrose content at 15 day after anthesis, GSSU25D is grain soluble sucrose content at 25 day after anthesis, GSSU35D is grain soluble sucrose content at 35 day after anthesis, *indicates the P < 0.05, ** indicates the P < 0.01, ns indicates no significant.

In this study, correlations between rice agronomic traits and yield were determined (**Figure 4**, correlation type: Spearman, exclude missing values (listwise)). The row and hill spacing configuration influence the accumulation of assimilates in individual rice plants and the population, thereby affecting the grain-filling process and the number of full grains, which ultimately impacts the rice yield and processing quality. The brown rice rate, milled rice rate, and head rice rate were negatively correlated with the number of productive panicles; positively correlated with the number of full grains, the seed setting rate, and the thousand-seed weight; and negatively correlated with the soluble sugar and soluble starch contents in leaves at the grain-filling stage, with some correlations reaching significant (p<0.05) levels. The chalkiness rate and chalkiness degree were negatively correlated with the soluble sugar and soluble starch contents in leaves at the grain-filling stage, both of which reached significant levels. The head rice yield was significantly (p<0.05) positively correlated with the head rice rate and extremely significantly (p<0.01) positively correlated with the soluble sugar and soluble starch contents at 25 and 35 DAF, indicating that the accumulation of assimilates at the late grain-filling stage is the key to increasing the head rice yield. In this study, the head rice yield of the WNDM method was the highest, and this method can be implemented to promote the rice yield and processing quality.

### 3.7 Nutritional quality of milled rice

The contents of protein and amylose are the main indicators for evaluating the nutritional quality of milled rice. A extremely significant Year × Mode interaction was observed for taste value (P < 0.01), indicating that the effect of planting configuration on taste value was not consistent across two years. The analysis of milled rice under the different plant spacing configurations (**Table 4**) revealed that the taste values under the LFM method were the highest, at 79.33 and 82.33. Except for being significantly (p<0.05) greater than that of the WNDM method in 2022, all the other values reached extremely significant levels. The protein content was highest under the NDM method in 2022 and highest under the CDM method in 2023, which was significantly (p<0.05) greater than that of the LFM method. The amylose content under the LFM method was significantly (p<0.05) greater than that under the NDM method. The difference in acid content between the NDM and WNDM methods reached extremely significant (p<0.01) level in 2022 and showed the same trend but no significant difference in 2023.

**Table 4:** Nutritional quality of milled rice.

Year	Method	Taste	Protein (%)	Amylose (%)	Acid (%)
2022	LFM	79.33 ± 0.38Aa	5.50 ± 0.29Ab	19.27 ± 0.23Aa	14.2 ± 0.27ABbc
	CDM	77.33 ± 0.63Bc	6.07 ± 0.18Aab	18.9 ± 0.43ABa	14.53 ± 0.24ABab
	NDM	77.00 ± 0.25Bc	6.37 ± 0.18Aa	18.27 ± 0.14Bb	14.90 ± 0.11Aa
	WNDM	78.33 ± 0.38ABb	6.17 ± 0.31Aa	19.47 ± 0.17Aa	13.83 ± 0.14Bc
2023	LFM	82.33 ± 0.80Aa	5.43 ± 0.67Ab	19.90 ± 0.25Aa	13.77 ± 0.33Aa
	CDM	77.00 ± 0.38Cc	6.50 ± 0.15Aa	18.70 ± 0.19Bc	14.17 ± 0.37Aa
	NDM	76.33 ± 0.38Cc	6.27 ± 0.37Aab	18.90 ± 0.29Bbc	14.20 ± 0.31Aa
	WNDM	79.00 ± 0.38Bb	5.83 ± 0.51Aab	19.33 ± 0.22ABb	13.93 ± 0.44Aa
	Y	*	ns	ns	ns
	M	**	**	**	ns
	Y×M	**	ns	ns	ns

Data in the table are means ± standard deviation, with three replicates. Different uppercase letters and lowercase letters indicate significant difference at P < 0.05 and P < 0.01 probability level within a column in a year, respectively. A two-way ANOVA was used to test the effects of mode, year, and their interactions. Y·indicates the year, M·indicates the mode,·Y × M·indicates the interaction between year and mode. * indicates the P < 0.05, ** indicates the P < 0.01, ns indicates no significant.

### 3.8 Characteristics of fine rice power and finely ground particles

**Figure 5:**
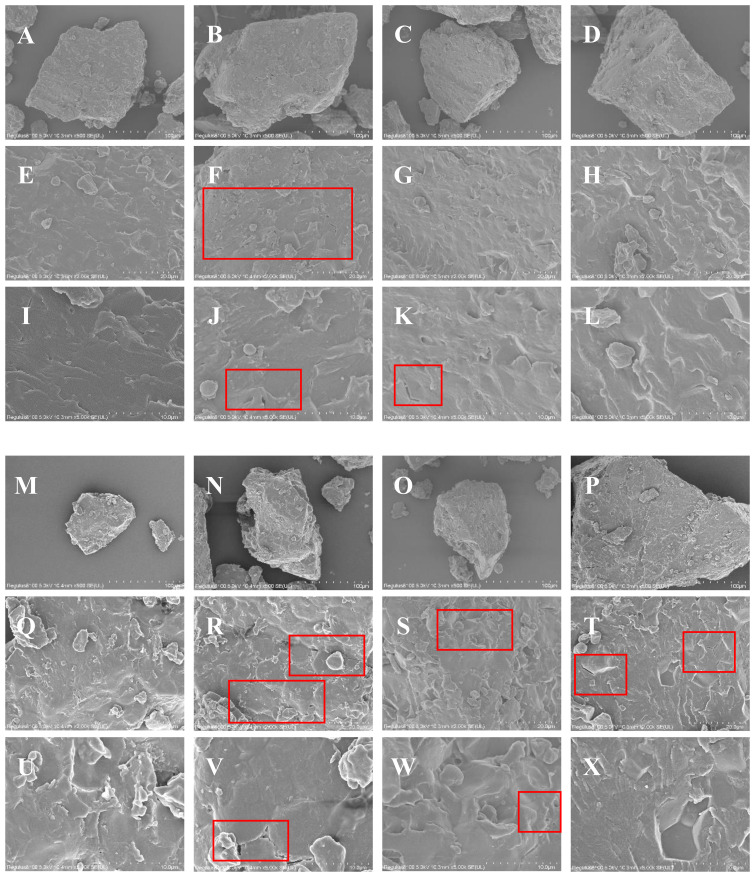
Morphological characteristics of rice flour under the different row and hill spacing configurations. **(A–D, M–P)** show scanning electron microscopy (SEM) images of rice flour particles from the LFM, CDM, NDM, and WNDM methods under 500 times magnification in 2022 and 2023, respectively; **(E–H, Q–T)** show SEM images of rice flour particles from the LFM, CDM, NDM, and WNDM methods under 2000 times magnification in 2022 and 2023, respectively; **(I–L, U–X)** show SEM images of rice flour particles from the LFM, CDM, NDM, and WNDM methods under 5000 times magnification in 2022 and 2023, respectively.

The characteristics of the fine rice powder particles indicated deterioration, with the starch particles exhibiting pores and wrinkles, resulting in a decrease in starch crystallinity. The analysis of the morphological characteristics of the fine rice powder under the different spacing configurations (**Figure 5**) revealed that the overall surface morphology of the fine rice powder in 2022 was smoother than that in 2023. The surface of the fine rice powder under the LFM method was relatively smooth, and the shape of starch particles was regular (**Figures 5A, E, I, M, Q, U**). The number of fine rice powder particles with a smooth surface increased under the CDM method, and pores were observed under 2000 and 5000 times magnification (**Figures 5F, J, R, V**), with wrinkles and cracks observed under 5000 times magnification in 2023 (**Figures 5J, V**). Under the NDM method, pores and cracks were observed under 5000 times magnification in 2022, and voids were observed under 5000 times magnification in 2023 (**Figure 5K**). The morphological characteristics of the fine rice powder under the WNDM method changed negligibly in 2022, and the surface was relatively smooth. Cracks and gaps were observed under 2000 times magnification (**Figure 5T**).

### 3.9 Quality and taste of cooking rice

The eating quality of cooking rice are important indicators for evaluating rice as a food. The Year × Mode interaction was not significant (P > 0.05) for all eating quality traits (Cooking taste value, Appearance, Hardness, Stickiness, Balance degree). The cooking and taste values of rice were highest under the LFM method, at 66.67 and 65.00, respectively, over two years, with no significant difference from those of the WNDM method but with significant differences from those of the CDM and NDM methods. The difference in appearance between the LFM and WNDM methods was no significant (p>0.05), but the values of both methods were significantly (p<0.05) greater than those of the CDM and NDM methods. The hardness of cooking rice under the CDM method was the highest, with values of 7.00 and 7.10 over two years period. In 2022, the hardness was significantly (p<0.05) higher than that of cooking rice under the other methods. The viscosity of cooking rice under the CDM method was the lowest and was significantly (p<0.05) lower than that of cooking rice under the LFM and WNDM methods in 2022. The balance value of cooking rice under the LFM method was the highest, significantly (p<0.05) higher than that of cooking rice under the CDM and NDM methods.

**Figure 6:**
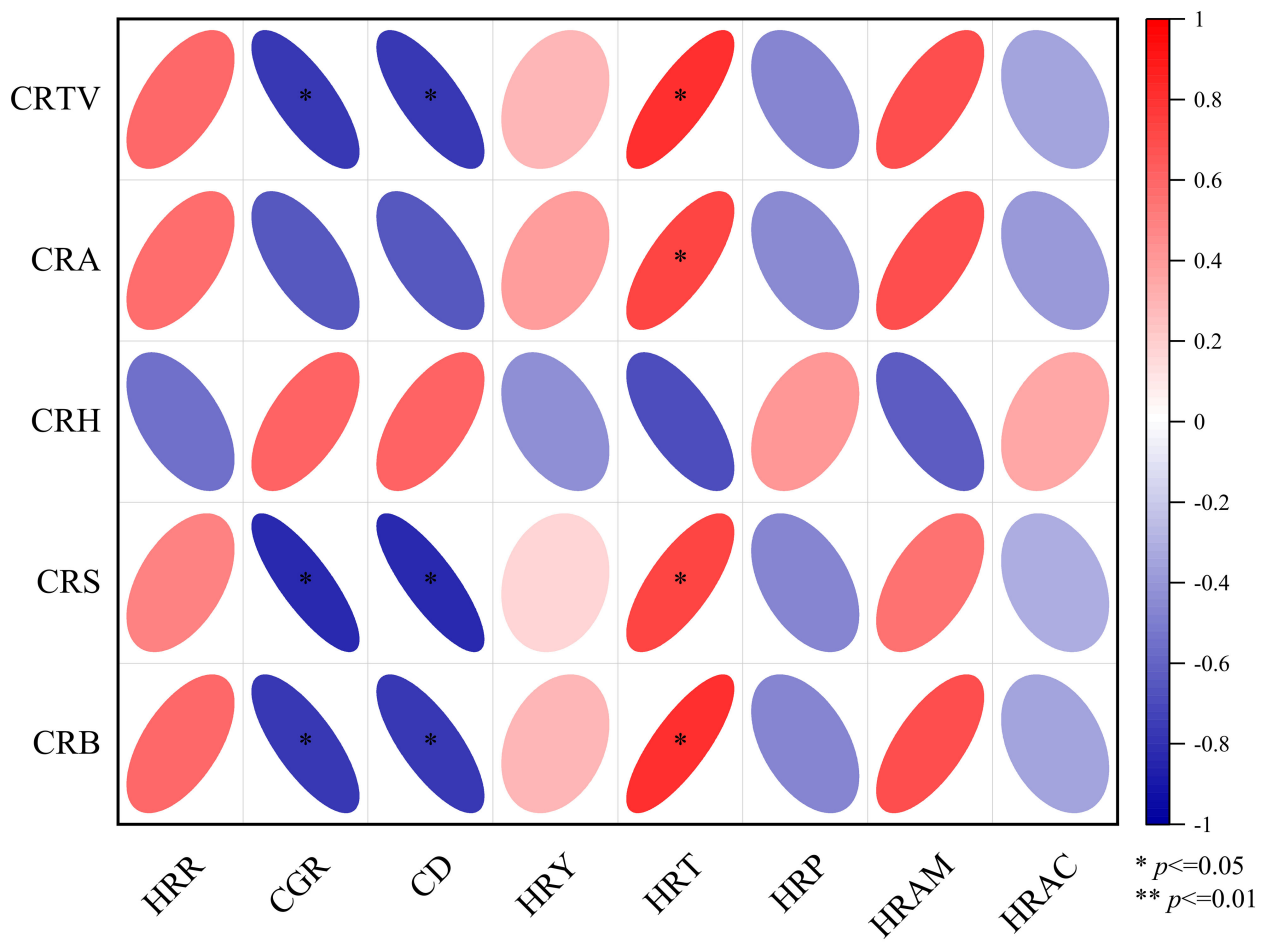
Cooking and processing quality correlation under the different plant spacing configurations. CRTV is cooking rice taste value, CRA is cooking rice appearance, CRH is cooking rice hardness, CRS is cooking rice stickiness, CRB is cooking rice balance degree, HRR is head rice rate, CGR is Chalky grain rate, CD is Chalkiness degree, HRY is head rice yield, HRT is head rice taste, HRP is head rice protein, HTAM is head rice amylose, HRAC is head rice acid.

The plant spacing configuration influences the starch accumulation characteristics and structural morphology of rice grains, which in turn affects the chalkiness rate and chalkiness degree of milled rice, ultimately significantly influencing the cooking and taste quality of rice (**Figure 6**). The cooking taste values were significantly (p<0.05) negatively correlated with the chalkiness rate and chalkiness degree and significantly (p<0.05) positively correlated with the taste value of milled rice. The appearance, viscosity, and balance of steamed rice were significantly positively correlated with the taste value of milled rice. High-quality rice grains are the foundation of high-taste-value steamed rice. Viscosity and balance were significantly (p<0.05) negatively correlated with the chalkiness rate and chalkiness degree. The hardness of cooking rice was positively correlated with the chalkiness rate and chalkiness degree, indicating that if the degree of grain filling is insufficient or not high, the number and degree of chalkiness grains increase. This leads to deterioration in the characteristics of finely ground rice particles, resulting in a decrease in starch crystallinity, which affects rice hardness and reduces the taste value.

## 4 Discussion

The rice yield and quality are comprehensively regulated by various genetic characteristics, ecological and environmental conditions, and cultivation management measures (Kakar et al., 2019; Chen et al., 2020, 2021). The plant spacing, as an important agronomic regulation method, alters the growth and development dynamics of rice plants by influencing the population structure and individual plants. This affects the accumulation and distribution of metabolites during grain filling, leading to differences in the grain setting rate and the thousand-grain weight at maturity, and ultimately significantly influencing rice yield and quality formation (Chen et al., 2020; Li et al., 2023).

### 4.1 Differences in rice yield formation caused by the plant spacing configuration

Numerous domestic and international scholars have confirmed that a suitable plant spacing configuration promotes yield improvement via the optimization of the structure and yield composition factors. Moreover, optimizing cultivation modes is an effective means to achieve yield potential (Peng et al., 2010, 2015; Wang et al., 2016) and narrow the yield gap between different ecological regions or varieties (Foley et al., 2011; Zheng et al., 2021). Li et al. (2015) reported that the implementation of artificial transplanting methods with spacings of 15 cm × 30 cm and 20 cm × 25 cm could increase the canopy production capacity and material accumulation while reducing the rice lodging rate and could effectively increase crop yields. Ruan et al. (2022) reported that appropriately increasing the transplanting density of rice to increase the number of productive panicles can fully manifest the positive effect of environmental resources on the rice yield and can serve as a sustainable development model for rice. Compared with the LFM treatment, the densification treatments significantly increased the number of stems in the population, and the number of productive panicles at maturity significantly increased across the various growth stages (**Tables 1**, **2**). This suggests that moderately increasing the planting density can effectively increase the number of productive panicles per unit area, providing a more sufficient panicle source foundation for yield formation, and is an important regulatory measure to increase rice yields. The canopy photosynthetic rate is an important criterion for evaluating whether crops can achieve high yields (Qin et al., 2025). A higher canopy photosynthetic rate before anthesis can result in greater photosynthetic assimilation, allowing nutrient organs in the internodes and sheaths to contain greater photosynthetic assimilation reserves. This can mobilize more dry matter back toward developing panicles (before anthesis) and grains (after anthesis), thereby increasing yields (Wang et al., 2025). Optimizing the plant spacing is beneficial for increasing biomass accumulation, which increases the accumulation of soluble sugars and soluble starch in grains after the full heading stage. In this study, the level of biomass accumulation under the various densification methods was significantly greater than that under the LFM method, providing a material basis for yield formation (**Figure 2**).

**Figure 7:**
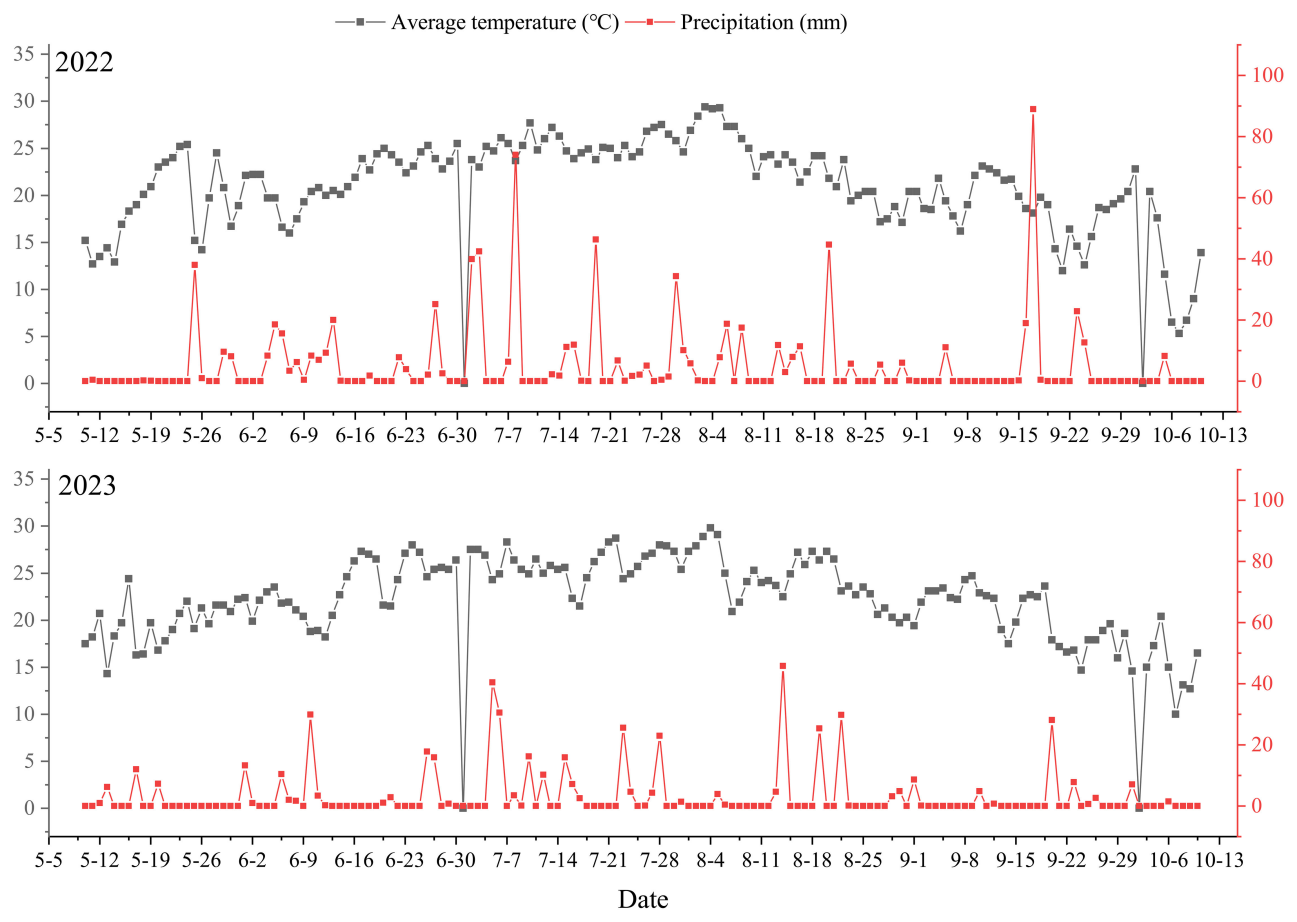
Meteorological data for the rice growing period at the experimental site in 2022 and 2023.

Dong et al. (2023) reported that the wide–narrow row spacing improved the population structure and roots physiological characteristics of grain filling stage, stabilized the number of full grains per panicle and thousand-grain weight, and increased the number of productive panicles, thereby increasing the rice yield. The results of this study indicated that, compared with the LFM treatment, all three densification configurations increased the rice yield (**Figure 7**), and the main contributing factor to yield improvement was the increase in the number of productive panicles (**Table 1**). This suggests that superior coordination—more panicles without a proportionate collapse in individual grain weight—is a hallmark of an optimized source-sink balance. The improved canopy ecology likely supports stronger root activity during grain filling, as noted by Dong et al. (2023), further sustaining the plant’s metabolic and nutrient uptake capacity during this crucial phase. Deng et al. (2022) and Dong et al. (2024) reported that the spatial structure configuration affects the canopy structure and the degree of population photosynthetic substance accumulation, leading to changes in the rice canopy microecology as well as changes in the rice grain-filling rate and the filling capacity at the later stage of rice growth. In this study, an increase in the number of productive panicles via the CDM and NDM methods resulted in a significant (p<0.05) decrease in the number of full grains per panicle and in the thousand-grain weight, thus making it difficult to fully exploit yield advantages (**Table 1**). The alternating wide and narrow rows create “light channels” that improve ventilation and light penetration into the canopy middle and lower layers. This is supported by our linked research (Dong et al., 2024), which showed that this structure maintains higher photosynthetic rates and delays senescence in flag and second leaves. This extended functional leaf lifespan directly translates into the enhanced late-stage assimilate supply observed in our metabolite data. Xiong et al. (2022) reported that the wide–narrow row high-efficiency planting mode, through the row spacing configuration of “compressing the middle and leaving both sides empty”, not only provides ventilated and transparent corridors between the wide rows but also provides a change in the illumination conditions from a two-dimensional regime to a three-dimensional regime, which optimizes the marginal advantages of the population, thereby increasing material accumulation and unleashing the potential yield advantages of the variety. In this study, the WNDM method increased the number of productive panicles compared with the LFM method and increased the number of full grains per panicle and the thousand-seeds weight compared with the CDM and NDM methods, resulting in a greater total output of grains per unit area and ultimately achieving the goal of increasing the yield (**Table 1**). Therefore, the optimization of the plant spacing configuration under the WNDM method increases the coordination of yield components and can serve as an effective agronomic regulation measure for achieving high and stable rice yields.

### 4.2 Differences in rice quality formation caused by the plant spacing configuration

While the optimized spatial configuration markedly enhanced yield through improved resource capture, its concurrent impact on grain quality necessitated an examination of how assimilate partitioning and stress modulation underpin these dual benefits. The photosynthetic compounds produced by the rice stem, sheath, leaves and other source organs are transported in the form of sucrose through the phloem to the grains and ultimately accumulate in the form of starch. Notably, the amount of transportation and unloading determines the processing and cooking quality of rice (Zhou et al., 2019; Li et al., 2019). Grain filling is influenced not only by internal factors such as variety genetics but also by external factors such as light and fertility. Wang et al. (2025) reported that the application of planting can increase the rapeseed yield by 11% and the resource utilization efficiency by 12.3% to 16.2% through the optimization of the field microenvironment and light–nitrogen matching conditions. The net photosynthetic rate of rice leaves in a population with a close plant spacing decreases, the number of photosynthetic products in individual leaves decreases, the ability to distribute assimilates decreases, endosperm starch synthesis is limited, the starch and amylose contents in grains decrease, the arrangement of starch bodies becomes loose, gaps increase, and the chalkiness rate and chalkiness degree of grains increase (Wang et al., 2021). In this study, compared with those of the LFM method, the chalkiness rate and chalkiness degree (**Table 3**) of the CDM and NDM methods significantly (p<0.05) increased, whereas the head rice rate decreased significantly (p<0.05). Notably, the characteristics of grains at stage I of morphogenesis (0–10 DAF), stage II of embryo enlargement (10–20 DAF), stage III of endosperm filling (20–30 DAF), and stage IV of grain maturation (30 DAF (mature)) differed (Tao et al., 2022). Therefore, the period of 15–35 days after the full heading stage is the most important stage for soluble sugar conversion and starch accumulation in grains. Our data on soluble sugar and starch accumulation provide a direct physiological explanation: at 25 and 35 days after flowering (DAF), WNDM plants sustained significantly higher levels of these non-structural carbohydrates in the grains than CDM and NDM (**Table 5**). This indicates a stronger and more prolonged “source” activity during the critical grain-filling period, which is essential for reducing chalkiness and improving milling recovery.

**Table 5:** Soluble sugar and starch accumulation after anthesis in leaves and grain.

Year	Method	Soluble starch in leaf (g/m^2^)			Soluble sugar in leaf (g/m^2^)			Soluble starch in grain (g/m^2^)			Soluble sugar in grain (g/m^2^)		
		15d	25d	35d	15d	25d	35d	15d	25d	35d	15d	25d	35d
2022	LFM	41.34 ± 0.80Bb	63.34 ± 1.52Bb	55.81 ± 1.46Bb	51.55 ± 1.00Bb	61.73 ± 1.48Ab	48.09 ± 1.26Bb	65.45 ± 1.25Bb	192.28 ± 4.01Bb	492.72 ± 10.68ABb	19.93 ± 0.38Bb	316.7 ± 6.63ABa	318.5 ± 6.94Ab
	CDM	43.90 ± 0.89ABa	70.63 ± 1.75Aa	63.7 ± 1.70Aa	56.16 ± 1.15Aa	67.09 ± 1.64Aa	57.19 ± 1.53Aa	76.58 ± 1.59Aa	192.38 ± 2.28Bb	478.05 ± 5.92Bb	23.76 ± 0.5Aa	296.25 ± 3.36Cb	292.18 ± 3.51Bc
	NDM	44.61 ± 0.84Aa	73.61 ± 1.45Aa	66.35 ± 1.40Aa	58.09 ± 1.07Aa	68.89 ± 1.37Aa	59.31 ± 1.24Aa	76.62 ± 1.42Aa	195.17 ± 4.02Bb	487.09 ± 10.53Bb	23.91 ± 0.44Aa	301.57 ± 6.34BCb	299.1 ± 6.56Bc
	WNDM	43.73 ± 0.85ABa	71.23 ± 1.82Aa	64.21 ± 1.76Aa	56.04 ± 1.07Aa	67.66 ± 1.75Aa	56.37 ± 1.55Aa	74.43 ± 1.42Aa	206.80 ± 2.27Aa	517.48 ± 6.08Aa	23.13 ± 0.44Aa	322.45 ± 3.66Aa	334.07 ± 3.89Aa
2023	LFM	41.89 ± 0.81Bc	66.68 ± 1.60Bb	56.58 ± 1.47Bb	53.42 ± 1.03Bc	62.85 ± 1.51Bb	45.93 ± 1.21Aa	69.5 ± 1.34Bb	200.25 ± 4.17ABb	506.8 ± 10.97ABb	21.56 ± 0.41Bb	339.47 ± 7.09ABa	366.23 ± 9.19Aa
	CDM	45.91 ± 0.94Aab	74.99 ± 1.85Aa	65.52 ± 1.74Aa	59.27 ± 1.22Aab	68.73 ± 1.69Aa	59.9 ± 1.63Aab	76.5 ± 1.57Aa	197.71 ± 2.31Bb	486.99 ± 5.97Bb	24.33 ± 0.5Aa	318.73 ± 3.62Cb	304.67 ± 0.59Bb
	NDM	47.56 ± 0.88Aa	77.52 ± 1.53Aa	68.2 ± 1.43Aa	61.46 ± 1.13Aa	71.48 ± 1.41Aa	62.19 ± 1.27Ab	76.18 ± 1.43Aa	199.30 ± 4.13Bb	489.34 ± 10.69Bb	23.77 ± 0.45Aa	321.29 ± 6.78BCb	311.75 ± 5.90Bb
	WNDM	45.01 ± 0.87Ab	75.16 ± 1.92Aa	64.66 ± 1.78Aa	57.92 ± 1.11Ab	69.16 ± 1.78Aa	58.31 ± 1.57Bc	77.03 ± 1.48Aa	211.50 ± 2.35Aa	529.54 ± 6.26Aa	23.99 ± 0.46Aa	350.80 ± 3.94Aa	356.65 ± 3.18Aa
	Y	**	**	ns	**	**	*	*	**	*	**	**	**
	M	**	**	**	**	**	**	**	**	**	**	**	**
	Y×M	ns	ns	ns	ns	ns	*	ns	ns	ns	ns	ns	**

Data in the table are means ± standard deviation, with three replicates. Different uppercase letters and lowercase letters indicate significant difference at P < 0.05 and P < 0.01 probability level within a column in a year, respectively. A two-way ANOVA was used to test the effects of mode, year, and their interactions. Y·indicates the year, M·indicates the mode,·Y × M·indicates the interaction between year and mode. * indicates the P < 0.05, ** indicates the P < 0.01, ns indicates no significant.

The starch content and distribution status of rice endosperm are closely related to soluble sugar metabolism process in the plant (Zhu et al., 2022; Ma et al., 2023; Li et al., 2024). Insufficient filling results in gaps caused by incomplete starch filling, leading to irregular shapes and uneven surfaces of finely ground starch particles. In this study, under the three densification methods, the surface of the fine ground powder particles changed from smooth to wrinkled with the appearance of pores, and the number of particles attached to the surface increased, indicating the accumulation of small particles (**Figure 5**). The CDM and NDM methods demonstrated the most significant (p<0.05) changes, which are related to the deterioration in starch granules caused by insufficient grain filling. Moreover, starch granules exhibited pores and wrinkles, leading to a decrease in starch crystallinity and affecting the eating quality of rice. In addition, the increase in protein content better mitigated the gaps caused by insufficient and incomplete starch filling (Noori et al., 2022), yielding a harder and tighter endosperm structure. Moreover, the increase in protein content limits water absorption during rice cooking, resulting in a decrease in the taste value. The protein content in grains under the different densification configuration methods was significantly (p<0.05) greater than that under the LFM method (**Table 4**). The additional protein can fill the pores in starch crystals, which is the reason for the increase in hardness and the decrease in cooking taste value under the CDM and NDM methods with respect to the cooking quality in this study. These results highlight that row and hill configuration is not merely a yield-enhancing tactic but a critical lever for quality-driven japonica rice cultivation, urging the integration of canopy architecture management into regional best practices to meet both market demands.

### 4.3 Differences in the plant spacing configuration for achieving an efficient food supply

The yield of rice is determined as the product of the number of productive panicles, the number of full grains per panicle, and the thousand seeds weight. After the harvest, rice is processed to produce milled rice, which is a staple food for humans (Zhou et al., 2019; Chen et al., 2023). Therefore, the effective grain supply is affected by both the rice yield and the milled rice rate. In this study, the densification methods all increased the rice yield but also reduced the milled rice rate and head rice rate to varying degrees. The head rice rate significantly decreased under the NDM method, and the advantage of an increased yield could not compensate for the disadvantage of a reduced head rice rate, resulting in a decrease in the final head rice yield, which is not conducive to ensuring an effective food supply of rice (**Figure 3**). WNDM maintained a head rice rate comparable to LFM and significantly higher than CDM and NDM, WNDM is not simply a yield-enhancing practice, but a strategy for quality-conscious intensification.

The source organs (leaves) of rice generate a large amount of carbon assimilates through photosynthesis, which are transported and distributed by the vascular system and stored in storage organs (grains), thereby resulting in yield accumulation (Jeena et al., 2019). Assimilates (sucrose) are transported over long distances through the phloem and eventually reach the storage end. Sucrose then passes through thin-walled cells and bead-like protrusions, enters the endosperm layer at the back of the grain, and finally reaches endosperm cells, thus affecting endosperm development (Yu et al., 2024). The plant spacing configuration affects the accumulation of biomass in different plant organs, and poor source–sink relationships can lead to imbalanced allocation of carbon assimilates, resulting in decreased yield and quality levels. In this study, the nutritional and cooking quality values of milled rice under the WNDM method were slightly lower than those of milled rice under the LFM method but still higher than those of milled rice under the CDM and NDM methods (**Table 6**). This is due to the accumulation of soluble starch and soluble sugars at 25 and 35 DAF, which provides a basis for determining the cooking quality. Moreover, the WNDM method demonstrated relatively stable cooking and nutritional quality values and provided the highest yield output. Thus, it was considered the best planting method for sustainable intensification of japonica rice systems, providing both a scientific framework to decode source-sink-environment interplay.

**Table 6:** Eating quality of cooking rice.

Year	Method	Cooking taste value	Appearance	Hardness	Stickiness	Balance degree
2022	LFM	66.67 ± 0.58Aa	6.07 ± 0.08Aa	6.53 ± 0.05Bc	5.90 ± 0.20Aa	6.07 ± 0.09Aa
	CDM	62.00 ± 1.00Bc	5.27 ± 0.13Bc	7.00 ± 0.09Aa	5.37 ± 0.25Ab	5.30 ± 0.14Bc
	NDM	64.00 ± 1.00Bb	5.67 ± 0.15ABb	6.77 ± 0.07ABb	5.63 ± 0.25Aab	5.70 ± 0.19ABb
	WNDM	66.33 ± 0.58Aa	6.00 ± 0.11Aa	6.57 ± 0.07Bc	5.90 ± 0.10Aa	6.00 ± 0.12Aab
2023	LFM	65.00 ± 1.32Aa	5.80 ± 0.22Aa	6.67 ± 0.29Aab	5.73 ± 0.20Aa	5.80 ± 0.27Aa
	CDM	61.00 ± 1.32Ac	5.23 ± 0.16Ab	7.10 ± 0.10Aa	5.33 ± 0.25Aa	5.23 ± 0.20Ab
	NDM	61.67 ± 1.04Abc	5.33 ± 0.16Ab	6.93 ± 0.22Aab	5.27 ± 0.21Aa	5.27 ± 0.22Ab
	WNDM	64.33 ± 1.04Aab	5.73 ± 0.11Aa	6.63 ± 0.07Ab	5.50 ± 0.23Aa	5.73 ± 0.16Aab
	Y	**	**	ns	*	ns
	M	**	**	**	*	**
	Y×M	ns	ns	ns	ns	ns

Data in the table are means ± standard deviation, with three replicates. Different uppercase letters and lowercase letters indicate significant difference at P < 0.05 and P < 0.01 probability level within a column in a year, respectively. A two-way ANOVA was used to test the effects of mode, year, and their interactions. Y·indicates the year, M·indicates the mode,·Y × M·indicates the interaction between year and mode. * indicates the P < 0.05, ** indicates the P < 0.01, ns indicates no significant.

## 5 Conclusions

In conclusion, our study demonstrates that the WNDM method is the optimal planting strategy for this region, primarily because it successfully optimizes the trade-off between grain yield and quality to maximize head rice yield. The key mechanism lies in its enhanced late-stage assimilate supply and superior coordination of yield components, which boosts productive panicle numbers and biomass accumulation. WNDM with optimized plant spacing is the recommended practice to increase regional yield and secure a high-efficiency food supply, despite a slight reduction in some quality parameters compared to LFM.

## Data Availability

The original contributions presented in the study are included in the article/**Supplementary Material**. Further inquiries can be directed to the corresponding author.
