# Supplementary figures and images for: Mechanized wide narrow row densification configuration enhances japonica rice yield and head rice quality in Northern China
Source: Front Plant Sci. 2026 Feb 4;17:1727060. doi: 10.3389/fpls.2026.1727060 (PMC12913112; doi:10.3389/fpls.2026.1727060)

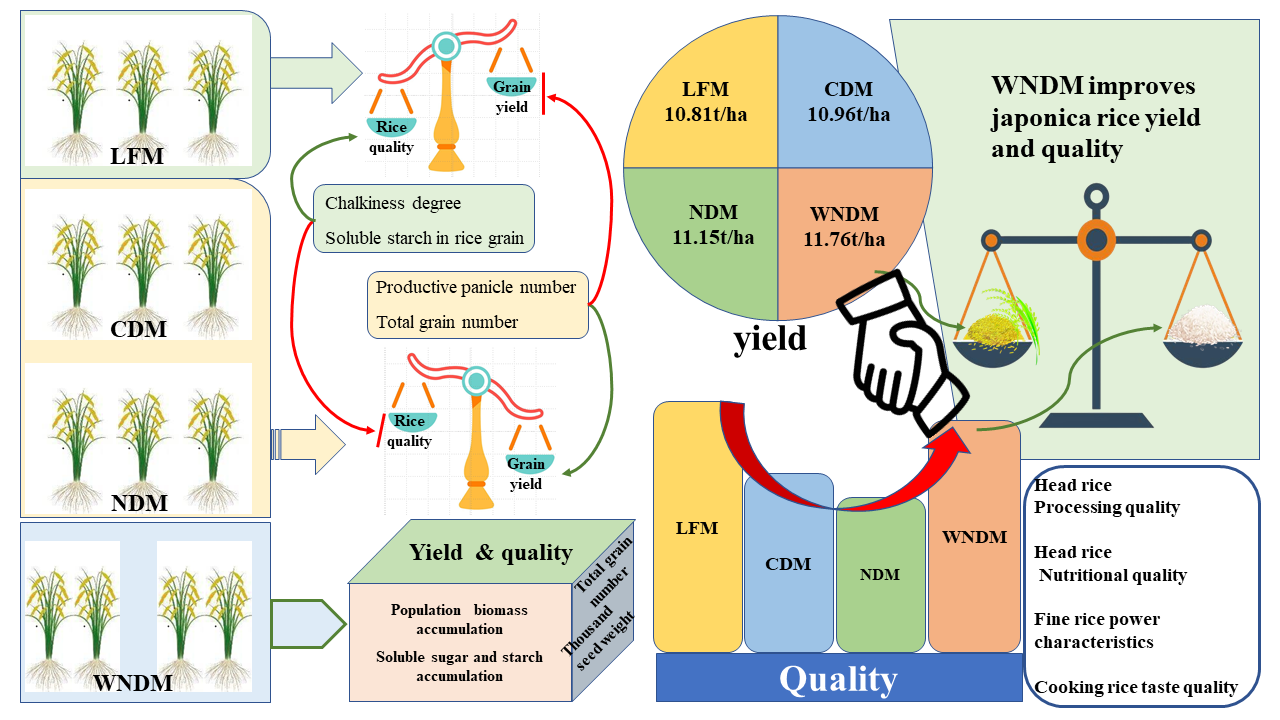

Supplement: Supplementary file 1 [file Image1.tif]
